# A predictive model for identifying secondary underlying diseases of hemophagocytic lymphohistiocytosis

**DOI:** 10.3389/fimmu.2023.1143181

**Published:** 2023-04-28

**Authors:** Wei-bo Gao, Li-juan Hu, Xiao-lu Ma, Mao-jing Shi, Chun-yu Wang, Yong Ma, Xiao-jing Song, Ji-hong Zhu, Tian-bing Wang

**Affiliations:** ^1^ Department of Emergency, Peking University People’s Hospital, Beijing, China; ^2^ Peking University People’s Hospital, Peking University Institute of Haematology, Beijing, China; ^3^ National Clinical Research Center for Haematologic Disease, Beijing, China; ^4^ Trauma Center, Peking University People’s Hospital, Beijing, China

**Keywords:** hemophagocytic lymphohistiocytosis, hematologic, rheumatic diseases, predictive model, secondary

## Abstract

**Background:**

Secondary hemophagocytic lymphohistiocytosis (HLH) is a rare, life-threatening disease of immune hyperactivation that arises in the context of infectious, inflammatory, or neoplastic triggers. The aim of this study was to establish a predictive model for the timely differential diagnosis of the original disease resulting in HLH by validating clinical and laboratory findings to further improve the efficacy of therapeutics for HLH.

**Methods:**

We retrospectively enrolled 175 secondary HLH patients in this study, including 92 patients with hematologic disease and 83 patients with rheumatic disease. The medical records of all identified patients were retrospectively reviewed and used to generate the predictive model. We also developed an early risk score using multivariate analysis weighted points proportional to the *β* regression coefficient values and calculated its sensitivity and specificity for the diagnosis of the original disease resulting in HLH.

**Results:**

The multivariate logistic analysis revealed that lower levels of hemoglobin and platelets (PLT), lower levels of ferritin, splenomegaly and Epstein−Barr virus (EBV) positivity were associated with hematologic disease, but young age and female sex were associated with rheumatic disease. The risk factors for HLH secondary to rheumatic diseases were female sex [OR 4.434 (95% CI, 1.889-10.407), *P* =0.001], younger age [OR 6.773 (95% CI, 2.706-16.952), *P*<0.001], higher PLT level [OR 6.674 (95% CI, 2.838-15.694), *P*<0.001], higher ferritin level [OR 5.269 (95% CI, 1.995-13.920), *P* =0.001], and EBV negativity [OR 27.656 (95% CI, 4.499-169.996), *P*<0.001]. The risk score included assessments of female sex, age, PLT count, ferritin level and EBV negativity, which can be used to predict HLH secondary to rheumatic diseases with an AUC of 0.844 (95% CI, 0.836~0.932).

**Conclusion:**

The established predictive model was designed to help clinicians diagnose the original disease resulting in secondary HLH during routine practice, which might be improve prognosis by enabling the timely treatment of the underlying disease.

## Introduction

Haemophagocytic lymphohistiocytosis (HLH) is a rare, life-threatening disease characterized by a dysregulated hyperinflammatory response associated with aberrant activation of lymphocytes and macrophages that results in hypercytokinemia (1–3). HLH can be broadly categorized into primary and secondary forms. Secondary HLH is usually associated with infectious, oncologic, rheumatologic, or other underlying causes (4–6). Many of the diagnostic features of this syndrome are nonspecific, and the combined clinical and laboratory findings resemble those of many diseases that might be triggers for HLH or that occur simultaneously with HLH (2, 5).

Acquired HLH has recently been increasingly recognized in patients with hematologic diseases and rheumatic diseases, but it is not easy to distinguish the two causes. Secondary HLH is a well-recognized complication of rheumatic diseases, such as systemic juvenile idiopathic arthritis (sJIA), adult-onset Still’s disease (AOSD), systemic lupus erythematosus (SLE), dermatomyositis, rheumatoid arthritis (RA), and Sjögren syndrome (7–12). In addition, secondary HLH in the setting of malignancy is commonly reported in adults and occurs in an estimated 1% of adult hematologic malignancies (13). Haematological malignancies and particularly lymphoma account for most cases of hematologic malignancy (HM)-related HLH (14). T-cell lymphoma is more commonly associated with the development of HLH (35%) than B-cell lymphoma (32%), and myeloid malignancies may also be complicated by HM-HLH. HM-HLH has the worst prognosis of all HLH subgroups, with a 5-year survival rate of less than 30% (14). Identifying complicated underlying diseases is an essential step because HLH therapy should be personalized according to the severity of the clinical evolution of the diseases and the underlying conditions (15).

Timely diagnosis of HLH is of special importance, as patients may be critically ill, and delays in diagnosis may result in poor outcomes (16). It is essential to identify the disorders underlying HLH and provide disorder-appropriate treatment to improve outcomes (17). Thus, once secondary HLH is diagnosed, the original disease resulting in secondary HLH should be identified as soon as possible. This study was performed to develop and validate a predictive model for the differential diagnosis of the underlying disease leading to secondary HLH and focused on the differential diagnoses of rheumatic or hematologic disease.

## Methods

### Patients

All patients diagnosed with secondary HLH between January 2012 and December 2021 at Peking University People’s Hospital were included in the current study. We then retrospectively reviewed the medical records of all patients that were identified. Familial HLH was excluded by an assessment of family history and due to the fact that familial HLH generally occurs at a younger age. Medical information was extracted from the records that addressed demographic characteristics (age, sex), clinical features (highest recorded temperature; presence of hepatomegaly, splenomegaly, or adenomegaly; earlier medical history; status of the diagnosis of hemophagocytic syndrome by the medical team caring for the patient as retained or not; treatment prescribed; underlying disease), laboratory findings (leukocyte and platelet counts, hemoglobin, liver enzyme, ferritin, triglyceride, cholesterol, fibrinogen, C-reactive protein, lactate dehydrogenase [LDH], blood urea nitrogen, creatinine, prothrombin time), and pathologic findings (hemophagocytosis on bone marrow aspiration or biopsy). All patients in our clinic with HLH had received bone marrow puncture, including bone marrow histological examination. In addition, lymph node biopsies were performed in patients with enlarged lymph nodes.

### Diagnostic criteria

The currently recognized diagnostic criteria for HLH, which were revised by the International Organization and Cell Association in 2004 (18), can be used to diagnose HLH if any of the following two criteria are met: (1) molecular diagnosis consistent with HLH or (2) fulfilment of the 5/8 criteria: 1. Fever temperature > 38.5°C for > 7 days, 2. Splenomegaly with the spleen tip palpated > 3 cm below the left costal margin, 3. Cytopenia involving 2 or more lines (hemoglobin < 90 g/L, platelets < 100*10^9^/L, or neutrophils <1.0*10^9^/L), 4. Hypertriglyceridemia and/or hypofibrinogenemia (fasting triglycerides ≥ 3.0 mmol/L, i.e., ≥ 265 mg/dl; fibrinogen ≤1.5 g/L), 5. Haemophagocytosis in bone marrow, spleen or lymph nodes (no evidence of malignancy); 6. Low or absent natural killer (NK)-cell activity, 7. Ferritin ≥500 mg/L, 8. Soluble CD25 (soluble IL-2 receptor) ≥ 2400 U/mL. Epstein−Barr virus (EBV) positivity was defined as an EBV nucleic acid quantitative DNA copy number exceeding 5*10^2^ copies/ml.

### Statistical methods

All statistical analyses were performed using SPSS software (version 26.0). The results are presented as medians and ranges for quantitative variables, as well as percentages for categorical variables. Fisher´s exact test and the chi-square test were used for comparisons between the groups for categorical variables; quantitative variables were compared with the Mann−Whitney U test and the Kruskal−Wallis test. We used a logistic regression model to determine risk factors.

Age, sex and variables with *P <*0.1 in the univariate analysis were included in binary multivariate logistic regression analysis to identify the risk factors for hematologic disease. The risk factors were assigned based on points weighted by the multivariate analysis as they relate to the *β*-regression coefficient values (which were rounded to the nearest integer). A linear transformation of the associated *β*-regression coefficient was used to score the risk factors (14, 16, 17); we then calculated a risk score for each patient. We also analyzed the areas under the receiver operating characteristic curve (AUCs) resulting from the analysis of the various risk factors to identify the sensitivity and specificity of analyses using these risk factors and risk scores. Statistical significance was identified using a two-sided *P <*0.05; IBM SPSS Statistics version 26.0 (IBM Corp., Armonk, NY, USA) was used for all statistical analyses.

## Results

### Patient characteristics

A total of 175 adult (≥16 years old) patients who were diagnosed with HLH were enrolled. There were 72 males (41.1%) and 103 females (58.9%). The age of all patients was reported in years, with a median age of 43 years (16 to 88 years). HLH was diagnosed as secondary to hematologic and rheumatic disease. Haematologic diseases were identified in 92 patients (52.5%), including lymphoma in 57 patients (62%), leukemia in 22 patients (23.9%), myelodysplastic syndrome in 8 patients (8.7%), and aplastic anemia in 2 patients (2.2%). A total of 83 cases of HLH (47.4%) were considered secondary to rheumatic diseases, including 33 cases (39.8%) of AOSD, 29 cases (34.9%) of systemic lupus erythematosus (SLE), 5 cases of dermatomyositis (6%), 3 cases of rheumatoid arthritis (3.6%), 2 cases of Sjögren’s syndrome (2.4%), and 2 cases of juvenile idiopathic arthritis (2.4%).

In total, 17.1% (30/175) of the patients had hepatomegaly, 57.1% (100/175) had splenomegaly, and 67.4% (118/175) had lymphadenopathy. In total, 18.3% (32/175) and 17.1% (30/175) of the patients had infections with cytomegalovirus (CMV) and Epstein-Barr virus (EBV), respectively. The incidence of hemophagocytic phenomena in the bone marrow was 40.6% (60/175). Finally, a total of forty-three patients died (24.6%) (**Table 1**).

**Table 1:** Demographic and clinical characteristics.

Characteristics	Total (n=175)	Rheumatic disease groups (n=83)	Haematologic disease groups (n=92)
Aetiological classification [n (%)]			
Lymphoma	57 (32.6)		57 (62.0)
Leukaemia	22 (12.6)		22 (23.9)
Myelodysplastic syndrome	8 (4.6)		8 (8.7)
Aplastic anaemia	2 (1.1)		2 (2.2)
Adult Still's disease	33 (18.9)	33 (39.8)	
Systemic lupus erythematosuserythematosus	29 (16.6)	29 (34.9)	
Dermatomyositis	5 (2.9)	5 (6.0)	
Rheumatoid arthritis	3 (1.7)	3 (3.6)	
Sjogren's syndrome	2 (1.1)	2 (2.4)	
Juvenile idiopathic arthritis	2 (1.1)	2 (2.4)	
Others	12 (6.9)	9 (10.8)	3 (3.3)
Female sex [n (%)]	103 (58.9)	65 (78.3)	38 (41.3)
Age [M (Q_1_,Q_3_),years]	43 (29,60)	35 (27,54)	54 (34,63)
Temperature [M (Q_1_,Q_3_), °C]	39.7 (39.0,40.0)	39.7 (39.2,40.2)	39.6 (39.0,40.0)
Hepatomegaly [n (%)]	30 (17.1)	6 (7.2)	24 (26.1)
Splenomegaly [n (%)]	100 (57.1)	40 (48.2)	60 (65.2)
Lymphadenopathy [n (%)]	118 (67.4)	52 (62.7)	66 (71.7)
WBC [M (Q_1_,Q_3_), ×10^9^/L]	1.82 (0.78,3.90)	2.50 (1.39,5.20)	1.30 (0.34,2.88)
NE [M (Q_1_,Q_3_), ×10^9^/L]	1.00 (0.22,2.25)	1.40 (0.62,3.10)	0.55 (0.05,1.50)
HBG [M (Q_1_,Q_3_), g/L]	69 (54,88)	76 (63,94)	60 (49,78)
PLT [M (Q_1_,Q_3_), ×10^9^/L]	32 (8,89)	67 (24,115)	12 (5,38)
ALT [M (Q_1_,Q_3_), U/L]	83 (36,243)	110 (40,365)	67 (34,144)
AST [M (Q_1_,Q_3_), U/L]	119 (50,268)	150 (62,448)	92 (44,221)
LDH [M (Q_1_,Q_3_), U/L]	864 (554,1510)	772 (555,1285)	983 (529,2025)
ALB [M (Q_1_,Q_3_), g/L]	27.0 (23.2,30.5)	26.4 (22.2,29.9)	27.6 (24.5,31.2)
TG [M (Q_1_,Q_3_), mmol/L]	3.05 (2.12,4.67)	2.79 (2.03,4.97)	3.11 (2.16,4.43)
TBIL [M (Q_1_,Q_3_), μmol/L]	20.2 (12.0,61.8)	14.8 (10.0,39.5)	26.9 (16.5,99.0)
DBIL [M (Q_1_,Q_3_), μmol/L]	11.0 (4.6,43.2)	5.7 (3.4,24.0)	14.1 (6.3,79.5)
PT [M (Q_1_,Q_3_), s]	14.4 (12.3,17.4)	13.4 (11.2,16.7)	15.3 (12.8,18.1)
APTT [M (Q_1_,Q_3_), s]	35.1 (30.1,41.9)	32.3 (28.1,40.8)	36.7 (32.0,42.5)
FIB [M (Q_1_,Q_3_), mg/dL]	149 (114,231)	153 (121,224)	148 (107,253)
FDP [M (Q_1_,Q_3_), μg/mL]	19.3 (10.5,56.3)	20.9 (9.7,57.0)	18.4 (10.7,55.9)
D-D [M (Q_1_,Q_3_), ng/dL]	3009 (1238,8189)	3607 (1256,9808)	2691 (1213,7786)
Ferritin [M (Q_1_,Q_3_), ×10^3^ ng/mL]	10.971 (3.276,30.561)	12.372 (3.050,40.814)	9.643 (3.740,22.479)
NK [M (Q_1_,Q_3_), %]	14.55 (13.48,15.91)	15.07 (12.71,16.58)	14.55 (13.78,15.63)
sCD25 [M (Q_1_,Q_3_), ×10^3^ pg/mL]	15.974 (10.000,24.000)	14.137 (8.324,23.333)	16.756 (12.564,33.949)
SUVmax	6.2 (4.0,9.6)	4.6 (3.3,6.6)	8.2 (5.5,12.4)
CMV positive [n (%)]	32 (18.3)	20 (24.1)	12 (13.0)
EBV positive [n (%)]	30 (17.1)	2 (2.4)	28 (30.4)
Haemophagocytic in BM (%)	40.6	42.7	38.8
HScore	234 (200,264)	234 (198,257)	235 (201,266)
Death [n (%)]	43 (24.6)	9 (10.8)	34 (37.0)

The clinical and laboratory findings of the total cohort, rheumatic and haematologic cohorts at the time of the initial assessments were summarized. WBC, white blood cell; NE, neutrophils; HBG, hemoglobin; PLT, platelet; ALT, alanine aminotransferase; AST, aspartate aminotransferase; ALB, albumin; LDH, lactate dehydrogenase; TG, triglycerides; TBIL, total bilirubin; DBIL, direct bilirubin; PT, prothrombin time; APTT, activated partial thromboplastin time; FIB, fibrinogen; FDP, Fibrinogen Degradation Products; D-D, d-dimer; NK, natural killer cell; CMV, cytomegalovirus; EBV, Epstein-Barr virus.

The first aspect of HLH treatment should focus on the treatment of original diseases. Glucocorticoid-based immunosuppressive agents were administered for the treatment of rheumatic disease-related HLH. A chemotherapy-based protocol was administered for the treatment of hematological disease-related HLH, such as HLH due to lymphoma. Second, supportive treatment, including blood transfusion support and anti-infection treatment, was also necessary for HLH treatment.

### Laboratory findings from the total cohort

The laboratory findings of the cohort at the time of the initial assessments are summarized in **Table 1**. The counts of three lineages of complete blood cells decreased. The leukocyte, neutrophil, hemoglobin, and platelet counts were calculated as 1.82 (0.78-3.9)×10^9^/L, 1.0 (0.22-2.25)×10^9^/L, 69 (54-88) g/L, and 32 (8-89) ×10^9^/L, respectively. The level of LDH was increased, with a median level of 864 U/L (554~1510). The median level of triglycerides was 3.05 mmol/L (2.12~4.67). The median level of fibrinogen was 149 mg/dL (114~231). The median level of D-dimer was 3009 ng/dL (1238~8189). The average serum ferritin level was 10.971 ×10^3^ ng/ml (3.276~30.561). The median level of NK cell activity and sCD25 levels were 14.55% (13.48~15.91) and 15.974 ×10^3^ pg/ml (10.000~24.000), respectively (**Tables 1**, **2**).

**Table 2:** Laboratory findings and the ratios in rheumatic and haematologic disease cohorts.

Characteristics [n (%)]	Total (n=175)	Rheumatic disease groups (n=83)	Haematologic disease groups (n=92)
Temperature>38.5 °C	162 (92.6%)	76 (91.6)	86 (93.5%)
WBC<4×10^9^/L	132 (75.4%)	54 (65.1%)	78 (84.8%)
NE<1×10^9^/L	87 (49.7%)	30 (36.1%)	57 (62.0%)
HBG<90g/L	134 (76.6%)	55 (66.3%)	79 (85.9%)
PLT<100×10^9^/L	140 (80.0%)	55 (66.3%)	85 (92.4%)
TG>3 mmol/L	89 (50.9%)	39 (47.0%)	50 (54.3%)
FIB<150 mg/dL	88 (50.3%)	41 (49.4%)	47 (51.1%)
Ferritin≥0.5×10^3^ ng/mL	173 (98.9%)	83 (100.0%)	90 (97.8%)
NK<15.11%	115 (65.7%)	49 (59.0%)	66 (71.7%)
sCD25≥6.400×10^3^ pg/mL	154 (88.0%)	70 (84.3%)	84 (91.3)

Haemophagocytic lymphohistiocytosis related diagnostic index of the total cohort, rheumatic and haematologic cohorts were summarized in terms of number (ratio). WBC, white blood cell; NE, neutrophils; HBG, hemoglobin; PLT, platelet; TG, triglycerides; FIB, fibrinogen; NK, natural killer cell.

### Comparison of patients with rheumatic and hematologic disease-related HLH

The comparison of the clinical characteristics between patients with rheumatic and hematologic disease-related HLH was performed by univariate analysis. There were significant differences between the two groups in terms of sex, age, splenomegaly, absolute neutrophil count, hemoglobin, PLT, sCD25 levels, PET-CT SUV max, and EBV positivity (all *P*<0.05). In the rheumatic disease group, there was a higher proportion of females (78.3% vs. 41.3%) with younger age (35 vs. 54). However, in the hematologic disease group, the patients had obvious reductions in the counts of three cell lineages, as shown in their complete blood counts, and a higher sCD25 level (16.7 vs. 14.1) and PET-CT SUV max (8.2 vs. 4.6), and there were more patients with EBV positivity (30.4% vs. 2%) and more patients with splenomegaly (65.2% vs. 48.2%).

There were no significant differences between the two groups in terms of temperature, LDH, TG, FIB, ferritin, or NK-cell activity (**Table 3**).

**Table 3:** The comparison of clinical characteristics between patients with rheumatic and haematologic disease-related HLH, in terms of value (range).

Item Characteristic	Haematologic disease groups (n=92)	Rheumatic disease groups (n=83)		*P*
Female sex [n (%)]	38 (41.3)	65 (78.3)	24.680	<0.001
Age [M (Q_1_,Q_3_),years]	54 (34,63)	35 (27,54)	3.449	0.001
Temperature [M (Q_1_,Q_3_), °C]	39.6 (39.0,40.0)	39.7 (39.2,40.2)	1.165	0.244
Splenomegaly [n (%)]	60 (65.2)	40 (48.2)	5.164	0.023
Lymphadenopathy [n (%)]	66 (71.7)	52 (62.7)	1.641	0.200
NE [M (Q_1_,Q_3_), ×10^9^/L]	0.55 (0.05,1.50)	1.40 (0.62,3.10)	3.912	<0.001
HBG [M (Q_1_,Q_3_), g/L]	60 (49,78)	76 (63,94)	3.981	<0.001
PLT [M (Q_1_,Q_3_), ×10^9^/L]	12 (5,38)	67 (24,115)	6.099	<0.001
LDH [M (Q_1_,Q_3_), U/L]	983 (529,2025)	772 (555,1285)	1.230	0.219
TG [M (Q_1_,Q_3_), mmol/L]	3.11 (2.16,4.43)	2.79 (2.03,4.97)	0.212	0.832
FIB [M (Q_1_,Q_3_), mg/dL]	148 (107,253)	153 (121,224)	0.013	0.989
Ferritin [M (Q_1_,Q_3_), ×10^3^ ng/mL]	9.643 (3.740,22.479)	12.372 (3.050,40.814)	1.081	0.280
NK [M (Q_1_,Q_3_), %]	14.55 (13.78,15.63)	15.07 (12.71,16.58)	1.393	0.164
sCD25 [M (Q_1_,Q_3_), ×10^3^ pg/mL]	16.756 (12.564,33.949)	14.137 (8.324,23.333)	2.948	0.003
SUVmax	8.2 (5.5,12.4)	4.6 (3.3,6.6)	4.049	<0.001
CMV positive [n (%)]	12 (13.0)	20 (24.1)	3.568	0.059
EBV positive [n (%)]	28 (30.4)	2 (2.4)	24.127	<0.001

### Risk factors and risk scores for distinguishing patients with rheumatic and hematological disease-related HLH

In the multivariate logistic analysis, lower levels of PLT, lower levels of ferritin, splenomegaly and EBV positivity were associated with hematologic diseases, but young age and female sex were associated with rheumatic diseases (**Table 4**).

**Table 4:** Multivariate Logisticlogistic analysis for distinguishing patients with rheumatic and haematological disease-related HLH.

Factors	β	Standard error	Wald χ^2^	*P*	*OR*(95% *CI*)
Female sex	1.427	0.441	10.492	0.001	4.166 (1.757~9.880)
Age	-0.044	0.013	11.072	0.001	0.957 (0.932~0.982)
NK	0.062	0.067	0.837	0.360	1.064 (0.932~1.214)
sCD25	-0.014	0.018	0.610	0.435	0.986 (0.953~1.021)
Splenomegaly	-0.888	0.436	4.155	0.042	0.411 (0.175~0.966)
NE	-0.088	0.066	1.755	0.185	0.916 (0.805~1.043)
HBG	0.025	0.012	4.631	0.031	1.025 (1.002~1.048)
PLT	0.011	0.005	5.251	0.022	1.011 (1.002~1.021)
Ferritin	0.024	0.008	8.884	0.003	1.024 (1.008~1.040)
EBV negative	3.896	0.961	16.428	0.000	49.210 (7.479~323.796)
Constant	-5.338	1.699	9.876	0.002	0.005

In the multivariate logistic analysis, lower levels of PLT, lower levels of ferritin, splenomegaly and EBV positivity were associated with haematologic diseases, but young age and female sex were associated with rheumatic diseases.

The risk factors for HLH secondary to rheumatic diseases were female sex [OR 4.434 (95% CI, 1.889-10.407), *P* =0.001], younger age [OR 6.773 (95% CI, 2.706-16.952), *P*<0.001], higher PLT level [OR 6.674 (95% CI, 2.838-15.694), *P*<0.001], higher ferritin level [OR 5.269 (95% CI, 1.995-13.920), *P* =0.001], and EBV-negative status [OR 27.656 (95% CI, 4.499-169.996), *P*<0.001]. The largest Youden index was used to determine the cut-off value, and the cut-off values for age, PLT count, and ferritin were 47 years old, 40 × 10^9^/L, and 19.583 × 10^3^ ng/ml, respectively.

### Prediction model

**Figure 1:**
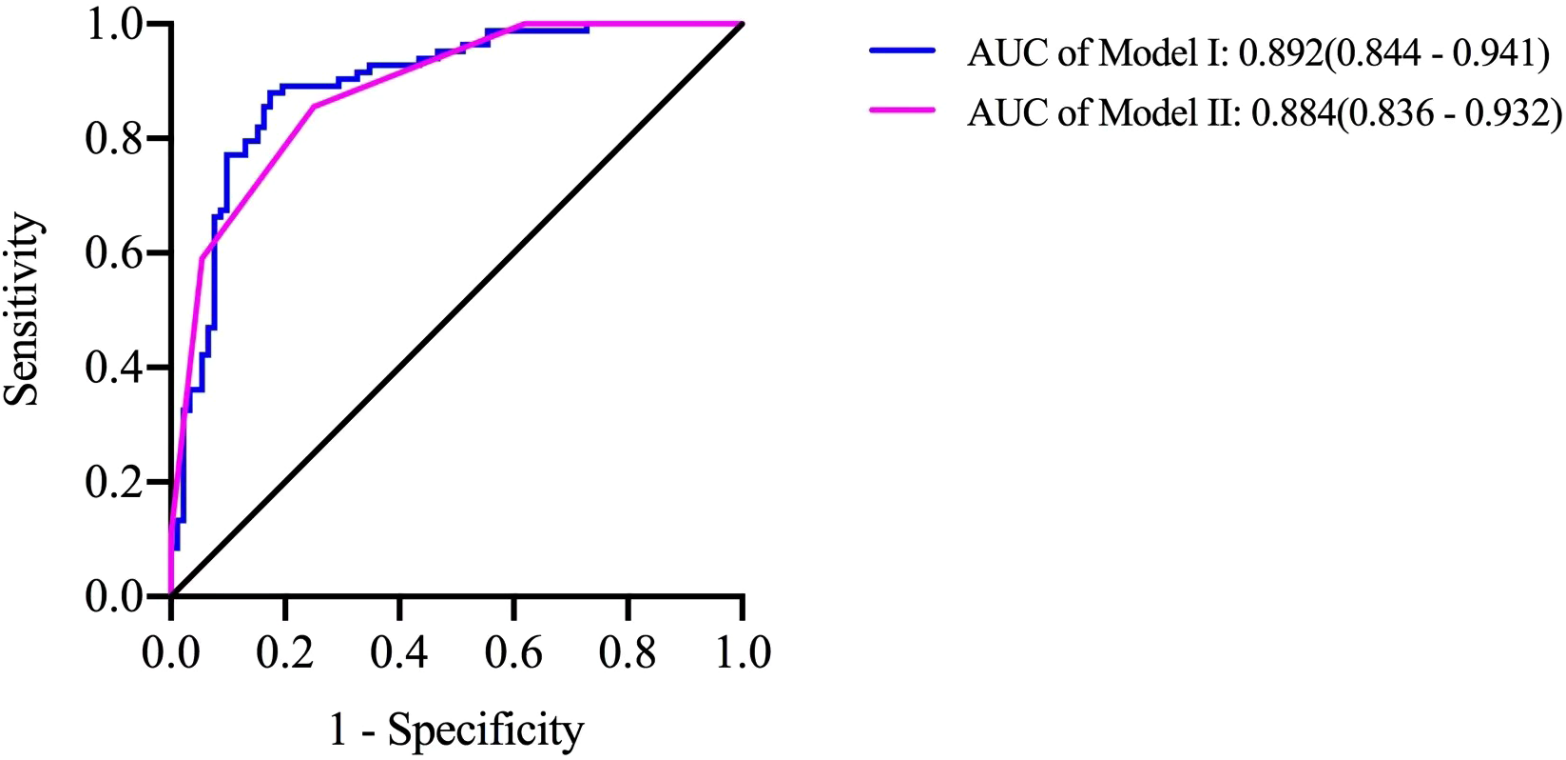
** **A predictive model for identifying diseases resulting in secondary HLH. Risk factors were used to produce the receiver operating characteristic curves Model I =1.427×Female-0.048×Age-0.820×Splenomegaly+0.024×HBG+0.009×PLT+0.022×Ferritin +3.810×EBV negativity-4.464; Model II =1×Female+1×Age+1×PLT+1×Ferritin +2×EBV negativity. When Model II ≥ 4, the sensitivity of predicting HLH secondary to rheumatic disease was 85.5% and the specificity was 75.0%.

The regression coefficient β value (Model I), which was rounded to the nearest integer (Model II, named SE), was used as the value of each risk factor to facilitate the clinical application of the predictive model; this scoring model, which is displayed in **Table 5**, was considered the gold standard, and Model II had a higher AUC of 0.844 (95% CI, 0.836~0.932), demonstrating sufficient predictive value. The maximum Youden index (when the score is 4 points) was set as the cut-off value of the SE model. Scores ≥ 4 suggested that HLH was secondary to rheumatic disease, while scores<4 suggested that HLH was secondary to hematological disease, with a sensitivity of 85.5% and a specificity of 75% (**Figure 1**).

**Table 5:** Multivariate logistic analysis of independent risk factors associated with immune or haematologichematologic diseases.

Factors	β	Assignment*	SE	Wald χ^2^	*P*	*OR* (95% *CI*)
Female	1.489	1	0.435	11.702	0.001	4.434 (1.889~10.407)
Age (<47 years)	1.913	1	0.468	16.696	<0.001	6.773 (2.706~16.952)
PLT (>40×10^9^/L)	1.898	1	0.436	18.931	<0.001	6.674 (2.838~15.694)
Ferritin (>19.583 ×10^3^ ng/mL)	1.662	1	0.496	11.242	0.001	5.269 (1.995~13.920)
EBV negative	3.320	2	0.927	12.839	<0.001	27.656 (4.499~169.996)
Constant	-6.410		1.136	31.824	<0.001	0.002

*Risk factors were scored using a linear transformation of the corresponding β-regression coefficient, female sex were was assigned as 1, age <47 years were ws assigned as 1, PLT>40×10^9^/L was assigned as 1, Ferritin >19.583×10^3^ ng/mL was assigned as 1, EBV negativity was assigned as 2. OR, odds ratio; CI, confidence interval. SE=1×Female+1×Age+1×PLT+1×Ferritin+2×EBV.

## Discussion

In the current study, a score system was established based on retrospective data obtained from patients with diagnosed HLH. The score comprised various clinical, biologic, and laboratory variables. The calculated prediction model focuses on the differential diagnosis of underlying disease causing HLH.

HLH is a complex disease that may affect many different organs. Therefore, the signs and symptoms may be extremely diverse, and no finding is pathognomonic for this condition (4, 19, 20). In this context, different sets of criteria have been suggested for helping clinicians diagnose the disease (1, 21). The identification of a simple model to make a differential diagnosis has been regarded as a necessary advance since HLH may be difficult to distinguish from an underlying disease (9, 22–25); this model would allow doctors to make adequate treatment decisions at the earliest opportunity.

Previously, Fardet et al. established and validated a reactive hemophagocytic syndrome diagnostic score (HScore) using a multicentre retrospective cohort of 312 patients (26). Finally, nine variables (three clinical [i.e., known underlying immunosuppression, high temperature, organomegaly], five biologic [i.e., triglyceride, ferritin, serum glutamic oxaloacetic transaminase, fibrinogen levels, cytopenia], and one cytologic [i.e., hemophagocytosis features on bone marrow aspirate]) were included in the HScore. However, the HScore was used for estimating an individual’s risk of having reactive hemophagocytic syndrome, not for the differential diagnosis of HLH or other underlying diseases.

A total of five variables were included in the model developed in this study, including sex (female = 1), age (younger than 47 = 1), platelet count (more than 40 = 1), ferritin level (more than 19583 = 1), and EBV status (negative =2). We found that a score of ≥ 4 was an indicator of a tendency to be diagnosed with rheumatic disease causing HLH. The epidemiological characteristics of rheumatoid disease include that it is more common among young women (27), which was consistent with our model. The ferritin level has not been widely used as an indicator for the differential diagnosis of HLH causes, and the most frequently observed conditions with markedly elevated ferritin include renal failure, hepatocellular injury, infections, rheumatic diseases or hematologic malignancies (28–31). A review of ferritin levels in pediatric patients used a cut-off of 10,000 mg/L and showed 90% sensitivity and 96% specificity for diagnosing HLH, but this was demonstrated in a small sample size (32). Another very recent study also concluded that ferritin levels > 10,000 mg/L were not specific for HLH (33). A report by Park et al., suggested that patients with a high serum ferritin level at the time of diagnosis showed a better prognosis (34). In our analysis, we found that a higher level of ferritin might be valuable for a potential diagnosis of rheumatic disease. Anemia and thrombocytopenia are more prevalent than neutropenia in HLH patients, and these symptoms usually develop early during the disease course. The challenge that clinicians face is to determine whether cytopenia is induced by HLH-related inflammation and hemophagocytosis as a manifestation of the underlying disease or by the treatment of the underlying disease triggering HLH (35, 36). Similarly, we calculated that lower levels of platelets were more common in patients with hematologic disease, indicating that thrombocytopenia might be due to primary marrow disorders. Clinicians can make timely judgements by applying this predictive model, which is beneficial to guide further treatment.

However, our prediction model also has limitations. The main concern is that it was developed using a retrospective study population from a single center. However, we will select data from other centers for model validation. Moreover, genetic tests related to familial HLH were not performed in our current analysis.

In conclusion, we have described the establishment of a predictive model that is designed to help clinicians diagnose the original disease resulting in secondary HLH in routine practice. Further research should evaluate the robustness of the model in other series of patients and should assess its performance in the diagnosis of the secondary form of HLH in a prospective cohort.

## Data availability statement

The raw data supporting the conclusions of this article will be made available by the authors, without undue reservation.

## Ethics statement

The studies involving human participants were reviewed and approved by the ethics committee of Peking University People’s Hospital. The patients/participants provided their written informed consent to participate in this study.

## Author contributions

W-BG, L-JH, J-HZ and T-BW designed the study. W-BG, L-JH, and X-LM analyzed the data and wrote the manuscript. All authors provided patient data and gave final approval for the manuscript.
